# Deepen the understanding and communication of animal models and experimental medicine research studies

**DOI:** 10.1002/ame2.12156

**Published:** 2021-03-12

**Authors:** Chuan Qin

**Affiliations:** ^1^ Chinese Association for Laboratory Animal Sciences Beijing China; ^2^ Institute of Laboratory Animal Sciences Chinese Academy of Medical Sciences & Peking Union Medical College Beijing China

At the first issue of the year 2021, I would like to extend my sincere gratitude for your continued support and attention to *Animal Models and Experimental Medicine* (*AMEM*) journal. On behalf of *AMEM*, I would like to wish you a Happy New Year and all the best!

*AMEM* is an international, open access academic journal dedicated to establish an international platform for academic exchange in the field of experimental animal sciences. *AMEM* is jointly founded by Chinese Association for Laboratory Animal Sciences (CALAS), Asian Federation of Laboratory Animal Science Associations (AFLAS), the International Council for Laboratory Animal Science (ICLAS) and Institute of Laboratory Animal Sciences (ILAS), Chinese Academy of Medical Sciences & Peking Union Medical College. Since its first publication in 2018, it has now entered into its fourth year.

*AMEM* has published four issues in 2020. Thirty‐nine papers were published on different contents, including animal models creation, experimental animal biotechnology, experimental animal welfare ethics, as well as infectious diseases, cardiovascular disease, neuroscience, and surgery in experimental animal models, drawing wide attention and citation. I would like to express my heartfelt thanks to all the readers, authors, and reviewers for their support and contribution, and to the editorial board and editing team for their efforts.

The year 2020 has been an extraordinary year, as the world, countries, and society have come together to face the global pandemic of COVID‐19. COVID‐19 is both a challenge and an opportunity, especially in the field of animal models and experimental medicine. Animal model studies are integral part of the scientific research, drug, and vaccine development for COVID‐19, as an important foundation for the treatment and prevention of the disease. Based on animal models, according to Koch's postulates, COVID‐19 pathogens and route of transmission were confirmed, rendering the virus infection, invasion, immune, and transmission process, which promotes the international scientific understanding of the disease. With the help of animal models, the effectiveness of various vaccines and medicines were evaluated, and it was also confirmed that antibodies produced after the first infection could protect the body from secondary infection.

Together with experts and authors, *AMEM* has published a series of animal models and experimental medicine studies on COVID‐19 under the precondition of strict review and rapid processing.^1^, ^2^ In 2021, we will continue to welcome submissions of animal studies on COVID‐19 to deepen the understanding and communication.

At present, *AMEM* has 7 Editor‐in‐Chief and executive Editor‐in‐Chief, 14 associate editors, and more than 40 experts in editorial board from China, the United States, Brazil, Israel, France, Australia, India, Thailand, and other countries, specialized in basic and clinical research studies in animal models and experimental medicine. We welcome more experts to join us to enhance academic integration and cooperation, and to put more impetus into the development of animal models and experimental medicine.

In 2021, we will continue to adhere to the original intention of animal models and experimental medicine research with a rigorous attitude of scientific research, efficient mode of operation, continue to promote research, communication and understanding of animal models, and experimental medicine.

Thank you once again for your support and trust. Looking forward to more communication and cooperation. I sincerely wish you a Happy New Year!
